# Preclinical Rationale, Clinical Efficacy, and Safety of the Selective AKT Kinase Inhibitor Capivasertib in Metastatic Hormone Receptor-Positive, Human Epidermal Growth Factor Receptor 2-Negative Breast Carcinoma: A Practical Narrative Review

**DOI:** 10.3390/curroncol33040198

**Published:** 2026-03-31

**Authors:** Maria Rosaria Valerio, Daniela Sambataro, Federica Martorana, Martina Greco, Chiara Mesi, Vittorio Gebbia, Paolo Vigneri, Giuseppa Scandurra

**Affiliations:** 1 Medical Oncology Unit, Policlinico, University of Palermo, 90100 Palermo, Italy; mariarosaria.valerio@unipa.it (M.R.V.); doc.martinagreco@gmail.com (M.G.); chiaramesi95@gmail.com (C.M.); 2 Medical Oncology, Ospedale Umberto I, 94100 Enna, Italy; daniela.sambataro@unikore.it; 3 Medical Oncology, University of Enna Kore, 94100 Enna, Italy; giuseppa.scandurra@unikore.it; 4 Medical Oncology, University of Catania, 95121 Catania, Italy; federica.martorana@unict.it (F.M.); paolo.vigneri@unict.it (P.V.); 5 Medical Oncology Unit, Humanitas Istituto Clinico Catanese, 95945 Misterbianco, Italy; 6 Medical Oncology Unit, CdC Torina, 90100 Palermo, Italy; 7 Medical Oncology Unit, Ospedale Cannizzaro, 95126 Catania, Italy

**Keywords:** capivasertib, fulvestrant, hormone positive breast cancer, AKT, PI3K, mTOR, PTEN

## Abstract

Most patients with advanced/metastatic hormone receptor-positive, HER2-negative breast cancer receive first-line therapy with cycline-dependent kinase 4/6 inhibitors plus endocrine therapy. Almost universally, these patients eventually progress due to the emergence of resistant cancer clones. Targeting the PIK3CA/AKT1/PTEN pathway is a way of overcoming resistance. Recently, the oral, selective AKT kinase inhibitor capivasertib has been approved for the treatment of estrogen receptor-positive/human HER2-growth factor receptor-2 advanced BC with alterations in PIK3CA/AKT1/PTEN, in combination with fulvestrant after progression on endocrine therapy. We performed a narrative review to recapitulate the available evidence about capivasertib in the management of advanced hormone receptor-positive, HER2-negative breast cancer, focusing on studies that address preclinical rationale, pharmacology, and clinically relevant problems.

## Simple Summary

Capivasertib is a selective AKT kinase inhibitor recently approved for the treatment of estrogen receptor-positive/HER2-negative advanced BC with alterations of the PIK3CA/AKT1/PTEN pathway, in combination with fulvestrant after progression on endocrine therapy. In this review we summarize preclinical evidence, pharmacology, clinically relevant problems, practical management and future options related to capivasertib.

## 1. Introduction

Approximately 70% of invasive breast carcinomas (BCs) display dependence on estrogens for cell proliferation and growth, by expressing estrogen and/or progesterone receptors (ER/PR) [1]. These BCs are referred to as hormone receptor-positive (HR+) tumors, as determined by immunohistochemistry and varying ER and PR staining intensities. Anti-hormonal agents are the mainstay of therapy for HR+/BC patients [2]. Such ER signaling pathway inhibitors include aromatase inhibitors (AIs; letrozole, anastrozole, exemestane), selective ER modulators (tamoxifen), and ER down-regulators (fulvestrant, FUL) used in daily practice. 

In the last decade, the availability of newer endocrine agents, such as cyclin-dependent kinase 4/6 inhibitors (CDK4/6i), and targeted therapies, has led to considerable improvements in survival rates in the adjuvant and advanced/metastatic settings [3]. Despite these progresses, most patients unfortunately present de novo or develop acquired resistance to ER-targeted therapies, leading to disease progression. Molecular alterations associated with resistance to CDK4/6i in HR+, HER2-negative BC include alterations in the phosphatidylinositol 3-kinase catalytic subunit alpha gene (PIK3CA), which regulates cell growth, division, survival, and metabolism, and mutations. PIK3CA activates the phosphatidylinositol 3-kinase/protein kinase B (AKT)/mammalian target of rapamycin signaling cascade, mutations of the Retinoblastoma gene (RB1), germline variants, dysfunction in fibroblast growth factor receptor signaling, FAT1 tumor suppressor gene loss, and the misregulation of cell cycle regulators like cyclin E and CDK2 [4,5,6]. The PI3K/AKT/mTOR pathway has been associated with stemness characteristics, proliferation, migration, epithelial to mesenchymal transition, and autophagy in cancer stem cells [4]. Thus, targeting PI3K/AKT/mTOR pathway can control the growth and proliferation of the BC stem cell population [7]. 

The serine/threonine kinase AKT plays a key role in the PI3K/AKT/mTOR signaling pathway, which regulates cellular metabolism, gene transcription, migration, proliferation, cell cycle, and survival [8,9]. Activation of AKT in ER+/HER2−/BC is correlated to resistance to endocrine therapy (ET) and carries a poor prognosis [8,9]. The most common AKT mutation is the E17K mutation, which causes a glutamic acid to lysine substitution. This mutation is present in 3–4% of BC and represents approximately 90% of the mutations in the AKT gene. The primary sources of AKT activation in cancers include mutations in the catalytic subunit of PI3K (PIK3CA), mutations in AKT1, activation of upstream signaling pathways, and loss of phosphatase/tensin homolog (PTEN) activity [9,10,11].

Recent research has shed light on mechanisms of resistance, showing that the PI3K/AKT/mTOR pathway is implicated in sustaining endocrine resistance [5,10,11]. This pathway has become the target of many new drugs for metastatic ER+/BC, and various classes of PI3K pathway inhibitors have been developed [8,11,12]. This review explores the significance of the PI3K/AKT/mTOR pathway in advanced ER+/HER2-BC, highlighting genetic contexts in which different inhibitors may be more effective. Additionally, we examine key trials evaluating drugs targeting this pathway and related pathways, and discuss the scientific rationale for developing triple combination therapies targeting ER, CDK4/6, and PI3K/AKT/mTOR in advanced/metastatic ER+/HER2−/BC.

## 2. Preclinical Data

**Figure 1:**
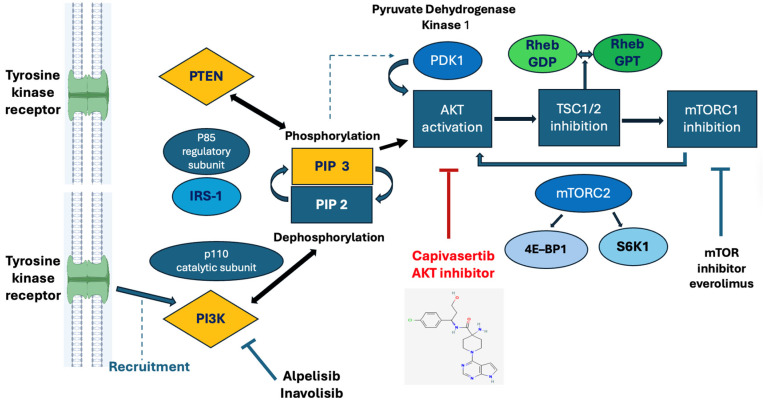
Membrane receptor tyrosine kinase stimulation recruits PI3K, inducing PIP2 phosphorylation to PIP3, and thus promoting AKT activation by PDK1.

As shown in Figure 1, the PI3K/AKT/mTOR pathway is a pivotal signaling cascade in BC, controlling cell growth, survival, and metabolism. Membrane tyrosine receptors recruit and activate PI3K, which activates the signaling cascade influencing PIP phosphorylation, recruitment of AKT to the membrane, and PDK1/mTORC2-mediated phosphorylation events. Upstream growth factors also stimulate PI3K, whose activation leads to the uncontrolled conversion of PIP2 to PIP3, which, in turn, recruits and activates protein kinase B (AKT). This process is also influenced by insulin receptor substrate-1 (IRS-1), a signaling adapter protein in humans encoded by the IRS1 gene [13]. It acts as a docking site, receiving inputs from the insulin receptor and playing a pivotal role in activating intracellular pathways that control cell growth, survival, and glucose metabolism, primarily by binding to PI3K to activate the AKT pathway and the Ras/MAPK pathway, thereby playing a vital role in maintaining metabolic balance and development. 

The phosphatase/tensin homolog (PTEN) counteracts this process, and the mTOR complexes regulate AKT activity both up- and downstream. PTEN is a tumor suppressor gene that physiologically inhibits this pathway by dephosphorylating phosphatidylinositol (3,4,5)-trisphosphate (PIP3), a second messenger in cells, thus converting PIP3 to PIP2 and activating AKT [13]. The latter inhibits the complexes of hamartin (TSC1) and tuberin (TSC2), as well as the mechanistic target of rapamycin complex 1 (mTORC1), which is downstream in the cascade. On the other hand, the mechanistic target of rapamycin complex 2 promotes the upstream AKT activation and influences 4E-BP1, i.e., the eukaryotic translation initiation factor 4E-binding protein 1. mTORC2 also stimulates the ribosomal protein S6 kinase beta-1 (S6K1), also known as p70S6 kinase, which is involved in the regulation of mitochondria morphology and function. This process is also influenced by Rheb (Ras homolog enriched in brain), which is a crucial small GTPase protein member of the Ras superfamily. It acts as a master switch, primarily by activating the mTORC1 pathway, a central regulator of cell growth, metabolism, protein synthesis, and autophagy, playing vital roles in normal cell function and BC, with its activity linked to nutrients and growth. AKT activation is also influenced by the mitochondrial enzyme pyruvate dehydrogenase kinase (PDK1), which regulates cellular metabolism by inactivating the pyruvate dehydrogenase (PDH) complex, thereby shifting glucose use from the Krebs cycle toward lactate production. This effector is crucial in cancer, promoting growth and chemoresistance by altering cellular energy pathways. Then, the mammalian target of rapamycin (mTOR) is activated, promoting downstream effects such as protein synthesis, cell growth, and inhibition of apoptosis, thereby increasing BC cell proliferation and survival. Upstream loss or mutations in the catalytic subunit alpha of the PIK3CA gene, which encodes phosphatidylinositol 3-kinase (PI3K), occur in 40% of cases. Mutations in AKT, such as AKT1 E17K, occurs in nearly 7% of cases of metastatic BC, leading to pathway hyperactivation, driving proliferation, drug resistance, especially to hormone/CDK4/6i therapies in HR+/HER2−/BC, and metastasis [13].

In ER+ BC, as acquired endocrine resistance develops, interactions between ER signaling and PI3K/AKT/mTOR signaling become more complex and interdependent [11,13]. In this context, inhibitors targeting PI3K, AKT, or mTOR can be used to counteract this dysregulation, often in combination with other agents [14,15]. In clinical practice, PIK3CA and mTOR inhibitors combined with ET therapy have shown clinical activity. Still, resistance to these drugs is common, likely due to a negative feedback pathway downstream of PI3K/AKT/mTOR [16]. In this context, direct targeting of AKT plus ET may improve BC treatment outcomes. Recently, a selective pan-AKT kinase inhibitor, capivasertib, has been developed [17].

## 3. PI3K/AKT/PTEN Molecular Testing

From a practical perspective, the identification of alterations in the PI3K/AKT/PTEN pathway relies on validated molecular diagnostic assays, several of which are approved as companion diagnostics in specific clinical settings. The most widely used assay for the detection of PIK3CA mutations is the FDA-approved therascreen^®^ PIK3CA RGQ PCR Kit, a real-time PCR test designed to detect hotspot mutations in exons 7, 9, and 20. This assay can be performed on formalin-fixed paraffin-embedded (FFPE) tumor tissue as well as on circulating tumor DNA (ctDNA) derived from plasma samples. In addition, next-generation sequencing (NGS)-based assays have also received FDA approval as companion diagnostics, including FoundationOne^®^ Liquid CDx, which enables the detection of PIK3CA mutations through plasma-based ctDNA analysis, and MI Cancer Seek, an NGS assay performed on tumor tissue that allows broader genomic profiling. In clinical practice, tissue-based testing remains the preferred approach when adequate tumor material is available, as it generally provides higher analytical sensitivity and allows simultaneous assessment of multiple genomic alterations when comprehensive NGS panels are used. However, liquid biopsy represents a valuable alternative when tumor tissue is unavailable or insufficient, or when a minimally invasive approach is preferred. Despite these advantages, both approaches present potential limitations. Tissue testing may be affected by tumor heterogeneity, sample quality, and limited tumor cellularity, while plasma-based assays may yield false-negative results in patients with low tumor burden or limited ctDNA shedding. Moreover, discordance between tissue and plasma results has been reported, reflecting both biological heterogeneity and differences in technical sensitivity across testing platforms. For these reasons, negative liquid biopsy results should be interpreted with caution and, when clinically indicated, confirmed with tissue testing whenever feasible. A clear understanding of the methodological characteristics and limitations of available companion diagnostic assays is therefore essential for optimal patient selection for therapies targeting the PI3K/AKT pathway.

## 4. Capivasertib

The selective AKT kinase inhibitor AZD5363 (capivasertib, CAP, Truqap^®^) is a novel oral drug approved for the treatment of ER+/HER2-negative advanced BC with alterations in PIK3CA/AKT1/PTEN, in combination with FUL, after progression on endocrine therapy (ET) [18].

*Molecular pathways*. This small molecule is a serine/threonine receptor kinase inhibitor that blocks the AKT signaling pathway, slowing tumor growth, and has been shown to halve the risk of disease progression compared to FUL alone, especially in tumors with alterations in the AKT1/PTEN gene [19]. CAP inhibits all three AKT isoforms (AKT1, 2, 3), which are crucial for tumor cell survival and proliferation, as well as the phosphorylation of downstream AKT substrates. In vitro, it inhibits the growth of cell lines from both hematological diseases and solid tumors, including BC cells, regardless of PIK3CA, AKT1, or PTEN mutations or alterations [20]. In vivo, CAP alone and in combination with FUL inhibited tumor growth in mouse xenograft models, including HR+/BC models with PIK3CA, AKT1, and PTEN alterations [13]. 

In BC cell lines adapted to long-term estrogen deprivation or tamoxifen, including MCF7, HCC1428, T47D, and ZR75.1, CAP has been shown to decrease cell proliferation in a dose-dependent manner, resensitize tumor cells to tamoxifen, and act synergistically with FUL [17]. 

At the molecular level, CAP reduced p-AKT/mTOR targets, which in turn reduced transcription mediated by the estrogen receptor alpha subunit (ERα) and concurrently reduced the recruitment of ER and cAMP response element-binding protein (CREB) to estrogen response elements on the TFF1, PGR, and GREB1 promoters [17]. Moreover, it decreased the expression of proteins that regulate the cell cycle. In a xenograft generated from an ER+ patient, adding FUL to CAP induced antiproliferative synergy and slowed tumor growth after treatment was stopped. CAP has also shown promising activity in combination with paclitaxel in cancer samples from patients with metastatic triple-negative BC harboring PI3K/AKT pathway alterations. Gris-Oliver et al. examined genetic and proteomic markers in 28 HER2-negative patient-derived xenografts (PDXs) treated with CAP alone or in combination with paclitaxel [16].

Mutations in PIK3CA/AKT1 and the absence of mTOR complex 1 (mTORC1)-activating alterations were associated with sensitivity to single-agent CAP, with sensitivity increasing from 64% to 89% when PTEN was excluded from the composite analysis. Additionally, low baseline pAKT S473 and residual pS6 S235 throughout treatment were associated with resistance, suggesting that parallel pathways avoid AKT/S6K1 activation in these mice. The data indicated two pathways of acquired resistance to CAP: loss of AKT1 p.E17K and overexpression of cyclin D1.

*Pharmacokinetic and pharmacodynamic*. CAP is administered orally [21]. Table 1 reports the main pharmacokinetic and pharmacodynamic characteristics of CAP. Neither high- or low-fat foods meaningfully alter CAP pharmacokinetics; therefore, it is recommended to take the drugs with a light meal. Exposure–response relationships were found for diarrhea (CTCAE Grades 2 to 4), rash (CTCAE Grades 2 to 4), and hyperglycemia (CTCAE Grades 3 or 4) at doses of 80 to 800 mg (0.2 to 2 times the approved recommended dosage); however, the time course of pharmacodynamic response and exposure–response relationship for the efficacy of CAP have not been fully characterized [13].

Zang et al. conducted a phase I trial evaluating the PK and safety of CAP, alone or in combination with paclitaxel, in 16 Chinese patients with advanced solid tumors [22]. CAP was rapidly absorbed and eliminated, with a half-life of approximately 10 h (geometric mean terminal elimination half-life of 9.7 h after a single dose). The most common adverse events were Grade 1–2 hyperglycemia, diarrhea, and rash. Notably, four (25.0%) patients achieved a confirmed partial response and four (25.0%) achieved stable disease.

*Formulations*. For patient convenience and production simplicity, CAP was first developed as a capsule and later as tablets. Dean et al. evaluated the PK comparability of both formulations (Part A) and the impact of meals on the PK and safety of the tablets (Part B) [23]. The tablet and capsule were similar in terms of PK and safety/tolerability. Food decreased the rate of absorption without appreciably affecting safety or tolerability, but it did not affect CAP bioavailability. The geometric least-squares mean ratios of tablet versus capsule for AUC τ and Cmax in 11 evaluable individuals were 0.90 (0.77–1.06) and 1.02 (0.86–1.20), respectively, indicating similar PK in the partly fasted state. Safety information for tablets and capsules was likewise identical. Compared with overnight fasting, tablet PK profiles showed a later tmax and lower Cmax after meals. The fasting- and fed-AUC τ and Cmax ratios were 0.67 (0.55–0.82) and 0.89 (0.76–1.05), respectively (*n* = 9). In both fed and fasted states, the tablet’s safety and tolerability profile was similar.

*Drug–drug interactions*. Miller et al. investigated the effect of a cytochrome P450 3A4 (CYP3A4) inhibitor itraconazole (ITZ) on the CAP pharmacokinetics [24]. In this phase I study, 11 healthy volunteers received a single dose of CAP 80 mg (stage 1), 4 doses of ITZ 200 mg over 3 days (stage 2), and a final dose of CAP 80 mg plus ITZ 200 mg (stage 3). Pharmacokinetics analysis showed that the coadministration of ITZ increased the Cmax and total exposure to CAP. The AUC from time of administration to infinity increased by 1.70-fold (90% CI 1.56–1.86) and 1.95-fold (90% CI 1.82–2.10), respectively.

*Dose identification*. Individual Bayes’ CAP steady-state exposure parameters (AUC, Cmax, and Cmin) were estimated by Fernandez et al. using a three-compartment population PK model [25]. There was no correlation between CAP exposure and either PFS or objective response rate in the CAPItello-291 study. However, AE probability, AE Grade ≥ 3, diarrhea AE Grade ≥ 2, and dosage modification were all associated with medication exposure. No noteworthy correlations were found between CAP exposure and adverse events leading to dosage cessation or significant adverse events. These findings corroborate the consistent benefit observed with aromatase inhibitor-resistant HR+/HER2−/BC on an intermittent CAP dosage schedule of 400 mg twice daily in conjunction with FUL, without dose modification based on body weight, age, race, or renal or hepatic function.

## 5. Phase I Trials

Phase I trials showed that heavily pretreated patients with tumors harboring activating PI3K pathway mutations who received CAP monotherapy had an objective overall response rate (ORR) below 30% [26,27,28]. Banerji et al. first reported a phase I, open-label study of CAP with different schedules showing that the dose-limiting toxicity was diarrhea for continuous administration, hyperglycemia for the intermittent 2-days-on/4-days-off schedule, and none for the 4-days-on/3-days-off schedule [26]. The maximally tolerated doses were 320, 480, and 640 mg for continuous, 4-days-on/3-days-off, and 2-days-on/4-days-off schedules, respectively. Dose-limiting toxicities were rash and diarrhea for the continuous schedule, hyperglycemia for the 2-days-on/4-days-off schedule, and none for the 2-days-on/4-days-off schedule. At the recommended phase II dose (480 mg bid, 4-days-on/3-days-off intermittent) CAP was well tolerated and achieved plasma levels and robust target modulation in tumors. Patients with *PIK3CA*-mutant BC and gynecologic cancer expansion showed a tumor shrinkage in 46% and 56% of cases, respectively (RECIST responses of 4% and 8%). 

In BC patients with AKT1 E17K-mutant tumors, who had undergone a median of five lines of previous treatment, the median progression-free survival (PFS) was 5.5 months (95% CI, 2.9 to 6.9 months) [27]. Biomarker analysis revealed an imbalance of the AKT1 E17K-mutant allele, predominantly due to copy-neutral loss-of-heterozygosity affecting the wild-type allele, which correlated with extended progression-free survival (hazard ratio [HR] 0.41; *p* = 0.04). Additionally, concurrent PI3K pathway hotspot mutations were associated with longer PFS (HR 0.21; *p* = 0.045). Persistent declines in AKT1 E17K in cfDNA were associated with improved PFS (HR 0.18; *p* = 0.004) and response (*p* = 0.025). The most common Grade ≥ 3 adverse events were hyperglycemia (24%), diarrhea (17%), and rash (15.5%). In a non-randomized basket trial by Kalinsky et al., 7 out of 15 patients with metastatic AKT1 E17K-mutated HR+/HER2−/BC achieved a partial response with CAP 480 mg, orally twice daily for 4 days on and 3 days off weekly every 28 days [28].

Smyth et al. conducted a phase I study (NCT01226316) to assess the safety, tolerability, and efficacy of CAP as a single agent or in combination with FUL in patients with AKT1 E17K-mutant ER+/BC identified by plasma BEAMing, droplet digital PCR, and next-generation sequencing [29]. In this trial, single-agent CAP achieved a 36% ORR in pretreated patients. CAP plus FUL achieved a 20% ORR in patients previously untreated with FUL. A reduction of at least 50% in AKT1 E17K at cycle 2 day 1 correlated with improved PFS. Combination therapy was associated with greater tolerability than monotherapy, as indicated by lower rates of Grade 3 or higher side effects, such as loose stools (5% versus 10%), cutaneous toxicity (9% versus 20%), and hyperglycemia (5% versus 30%). Data showed that CAP exerts a clinically meaningful activity in heavily pretreated women with ER+BC harboring AKT1 E17K-mutations despite progression to previous treatments, including FUL. Both tolerability and activity appeared enhanced with combination therapy. At expected therapeutic concentrations, CAP did not show clinically significant QT interval prolongation [30].

## 6. Phase II–III Trials

Table 2 shows the main published studies of CAP in advanced/metastatic HR+/HER2−/BC. A randomized, multicenter, double-blind, placebo-controlled, phase 2 trial (FAKTION) recruited 140 postmenopausal adult women with inoperable advanced or metastatic ER+/HER2−/BC with an ECOG performance status of 0–2 who had relapsed or progressed after AI to receive CAP/FUL or placebo/FUL with median PFS (mPFS) as the primary endpoint (one-sided alpha of 0.20) according to an intent-to-treat fashion [31,32]. Patients were 1:1 randomized to receive intramuscular FUL 500 mg on day 1 every 28 days (plus a loading dose on day 15 of cycle 1) with either CAP 400 mg (*n* = 69) or placebo (*n* = 71), orally b.i.d. on an intermittent weekly schedule of 4 days on and 3 days off (starting on cycle 1 day 15) until disease progression, unacceptable toxicity, loss to follow-up, or withdrawal of consent. Patients were stratified using an interactive web-based response system, minimizing measurable or non-measurable disease, primary or secondary AI resistance, PIK3CA status, and PTEN status. Median PFS reached 10.3 months (95% CI 5.0–13.2) in the group receiving CAP/FUL compared with 4.8 months (95% CI 3.1–7.7) for the placebo/FUL (HR 0.56; 95% CI 0.38–0.81; *p* = 0.0023). The difference was statistically significant (two-sided *p* = 0.0044; one-sided log rank test *p* = 0.0018). The secondary endpoint was median OS, which was 29.3 months (95% CI 23.7–39.0) in the CAP group and 23.4 months (95% CI 18.7–32.7) in the placebo group (HR 0.66; 95% CI 0.45–0.97; *p* = 0.035). Molecular analysis identified 76 mutated patients, which showed a mPFS of 12.8 months and 4.6 months (HR 0.44; 95% CI 0.26–0.72; *p* = 0.0014) and an OS of 38.9 months (95% CI 23.3–50.7) and 20.0 months (95% CI 14.8–31.4) for the CAP and the placebo groups (HR 0.46; 95% CI 0.27–0.79; *p* = 0.0047). By contrast, there were no statistically significant differences in PFS or OS in the non-altered subgroup treated with CAP (*n* = 30) versus placebo (*n* = 34). The most common Grade 3–4 adverse events included hypertension in 32% and 24% of patients in the CAP and the placebo groups, respectively. Diarrhea was reported in 14% of patients treated with CAP versus 4% in the placebo group, rash in 20% versus nil, infection in 6% versus 3%, and fatigue in 1% versus 4%. Severe adverse reactions occurred only in the CAP group. These included two cases of acute renal damage, three cases of diarrhea, two cases of rash, one instance of hyperglycemia, one case of loss of consciousness, one case of sepsis, and one case of vomiting. One death from an unusual lung infection was thought to be potentially connected to CAP therapy. The remaining fatalities in both groups (19 in the CAP group and 31 in the placebo group) were all due to illness, with one additional death in the CAP group having an unexplained cause. Despite the benefit of CAP-based therapy appearing to be independent of the PI3K/AKT/phosphatase and PTEN pathway alteration status, biomarker testing suggests that CAP was predominantly efficacious in patients with PI3K/AKT/PTEN pathway-altered tumors [32]. 

Chinese researchers also explored the efficacy and safety of CAP/FUL versus placebo/FUL in a prespecified two-stage design study (first stage: *n* = 24; second stage: *n* = 110) [36]. In the whole population, mPFS was 6.9 months (95% CI 5.4–9.2) in the CAP/FUL group and 2.8 months (95% CI 1.9–3.9) in the placebo-FUL group (HR 0.51, 95% CI 0.34–0.76) [36]. In patients with PIK3CA/AKT1/PTEN-altered tumors, mPFS was 5.7 months (95% CI 3.8–8.0), compared with 1.9 months (95% CI 1.8–3.7) in the control group (HR 0.41, 95% CI 0.19–0.85), and in PIK3CA/AKT1/PTEN-non-mutated tumors. The most frequent adverse events with capivasertib/FUL were diarrhea (60.6% versus 11.3% with placebo/FUL) and hyperglycemia (57.7% versus 17.7%), leading to discontinuation rates of 11.3% and 3.2% in the CAP and placebo arms, respectively. The benefit–risk profile of CAP/FUL in the Chinese cohort was favorable.

In 2023, the US Food and Drug Administration approved CAP in combination with FUL for adult patients with locally advanced/metastatic HR+/HER2−/BC who had received at least one prior endocrine therapy and harbored one or more alterations in the PIK3CA/AKT1/PTEN pathways, as detected by an FDA-approved test [19]. In 2025, the National Institute for Health and Care Excellence and the European Medicines Agency also approved CAP plus FUL [40,41].

*CAPITello-291 trial*. CAP approval was based on the phase III, prospective, randomized CAPITello-291 study (NCT04305496), which showed a statistically significant doubling of PFS, the principal study endpoint, in the overall population and in the PIK3CA/AKT1/PTEN-mutated ER+/HER2−/BC patient population treated with CAP/FUL or placebo/FUL (3.1 versus 7.3 months) [33,34]. The study was a randomized, double-blind, multicenter trial of 708 patients with advanced/metastatic HR+/HER2−/BC, including 289 patients (40.8%) with PIK3CA/AKT1/PTEN tumor alterations [33,34]. Pre-, peri-, and postmenopausal women and men with progressive disease after AI therapy, with or without prior CDK4/6i, were randomly assigned 1:1 to receive oral CAP 400 mg twice daily (twice daily for 4 days, followed by 3 days off) per week, with intramuscular FUL 500 mg (every 14 days for the first three injections, then every 28 days) (*n* = 355) or placebo (*n* = 353) with matching FUL dosing. Random assignment included stratification by the presence of liver metastases, previous treatment with CDK4/6i (691 patients), and geographical region. The dual primary endpoint was investigator-assessed PFS in the overall population and among patients with AKT pathway-altered (PIK3CA, AKT1, or PTEN) tumors. The overall population showed a statistically significant PFS benefit, with mPFS of 7.2 months in the CAP/FUL arm compared with 3.6 months in the placebo/FUL arm (HR 0.60; 95% CI 0.51–0.71; *p* < 0.001). However, these results were driven by 289 patients in the AKT pathway-altered population; the mPFS was 7.3 and 3.1 months in the CAP/FUL and placebo/FUL groups, respectively (HR 0.50; 95% CI 0.38–0.65; *p* < 0.001). An exploratory analysis of investigator-assessed PFS in the 313 (44%) patients in the biomarker-negative population showed uncertain benefit (HR 0.78; 95% CI 0.60–1.01).

Data were also confirmed in the 78 Japanese patients randomized in CAPItello-291 in which PFS numerically favored the CAP/FUL arm (HR 0.73; 95% CI 0.40–1.28), consistent with the analysis of PFS in the global population [35]. PFS was more favorable also in patients with PIK3CA/AKT1/PTEN-altered tumors treated with CAP/FUL (HR 0.65; 95% CI 0.29–1.39. The safety profile was not different to that in the global population. A comprehensive review and meta-analysis, presented as an abstract and including three trials with a total of 848 patients, shown that CAP substantially enhanced progression-free survival (PFS) (HR 0.61; 95% CI: 0.48–0.77, *p* = 0.0124) and overall survival (OS) (HR 0.68; 95% CI: 0.54–0.87, *p* = 0.0209) in advanced HR+/HER2−/breast cancer [39]. Treatment did not significantly raise the risk of fatigue (RR 1.37; 95% CI: 0.96–1.95, *p* = 0.0848), but it was linked to an elevated risk of diarrhea (RR 2.97; 95% CI: 2.27–3.87, *p* < 0.0001) and rash (RR 3.49; 95% CI: 2.15–5.65, *p* < 0.0001).

*Safety profile*. Tolerability was reported as acceptable, with no evidence of a detrimental effect on OS; however, adverse events leading to discontinuation occurred in 13.0% of patients receiving CAP and in 2.3% of those receiving placebo [33,34]. By contrast, the benefit-to-risk ratio was unfavorable in the biomarker-negative population. Severe grade 3 toxicity was more frequent with CAP, including hyperglycemia (18% all-grade, 2.8% grade ≥ 3), cutaneous toxicity (58% all-grade, 17% grade ≥ 3), and diarrhea (72% all-grade, 9% grade ≥ 3), rash (in 12.1% of patients, versus 0.3% of those receiving placebo/FUL) and diarrhea (in 9.3% versus 0.3%). 

*Management guidance*. Recently, clinical monitoring and management guidelines reported detailed information on CAP-based therapy management guidance and optimizing clinical benefit [42,43]. Frequent any-grade AEs with capivasertib/FUL “were diarrhea (72.4%), rash (38.0%), and nausea (34.6%); frequent grade ≥ 3 AEs were rash (12.1%), diarrhea (9.3%), and hyperglycemia (2.3%). Diarrhea, rash, and hyperglycemia occurred shortly after starting CAP/FUL [median days to onset (interquartile range) of any grade: 8 (2–22), 12 (10–15), and 15 (1–51), respectively] and were managed with supportive medications, dose reductions, interruptions, and/or discontinuation. Discontinuation rates were 2.0%, 4.5%, and 0.3%, respectively”. 

**Figure 2:**
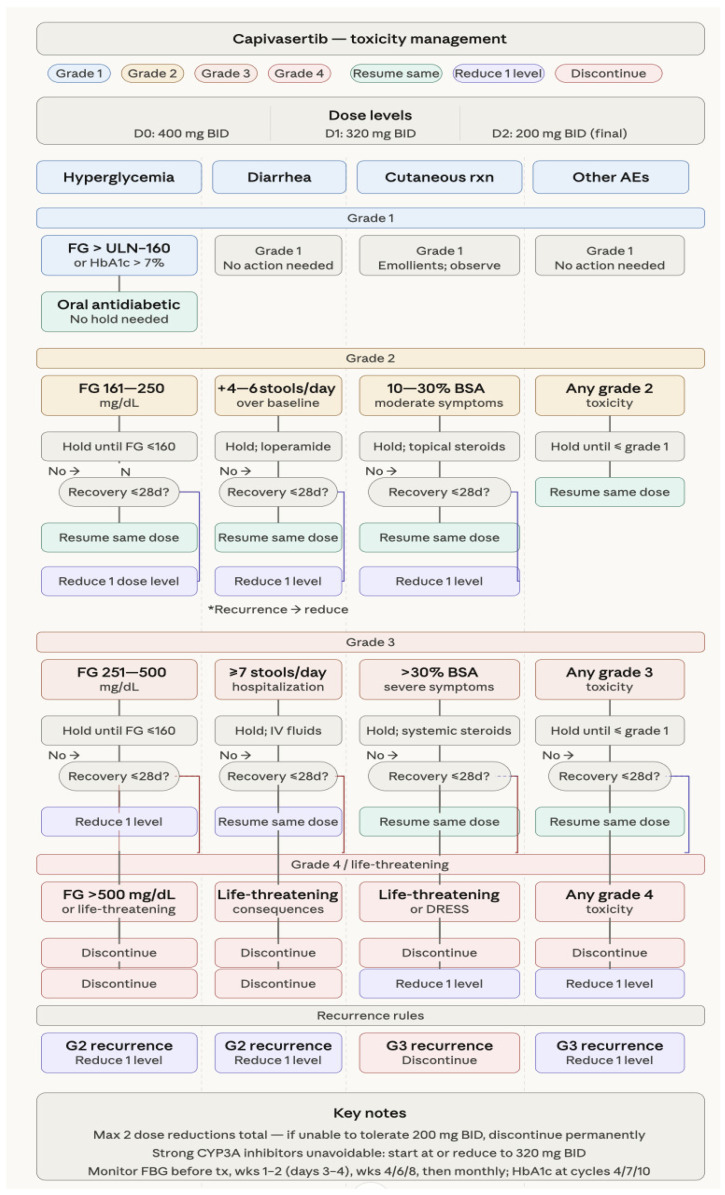
Toxicity management and suggested dose modifications.

Figure 2 shows toxicity management and suggested dose modifications. Hyperglycemia is a known adverse event associated with CAP and can occasionally lead to severe conditions such as diabetic ketoacidosis, which may be related to a pharmacodynamic interaction between CAP and glucose metabolism [44,45]. The incidence of Grade 3/4 hyperglycemia was 2.3% in the CAPItello-291 study, which excluded patients with diabetes on insulin or hemoglobin A1c > 8.0%. In Japan, a fatal instance of DKA during the first stages of capivasertib therapy has been documented. The best approach for glycemic management is still unclear, despite the knowledge that risk factors for severe hyperglycemia include obesity, a history of diabetes, and baseline blood glucose levels in the diabetic or pre-diabetic range. The median onset of hyperglycemia is 15 days, even with initially normal levels, and may require insulin administration. Standard hemoglobin A1c monitoring may fail to capture blood glucose fluctuations, suggesting caution in patients with metabolic risk factors. The putative mechanism underlying hyperglycemia is the inhibition of the intracellular insulin response, followed by promotion of glycogenolysis in the liver and down-regulation of AKT2’s ability to regulate glucose transport in adipose tissue, which can rapidly lead to diabetic ketoacidosis if untreated. 

Skin rash, affecting up to 38% of patients, typically starts within the first two weeks of treatment and is severe in 12% of cases. These often appear in the first two weeks as red, itchy, or peeling skin, sometimes resembling hand–foot syndrome. Immediate medical attention is required, as severe reactions (e.g., Stevens–Johnson syndrome, or drug reaction with eosinophilia and systemic symptoms—DRESS) can occur. Rashes can appear as itchy, red, or discolored bumps (maculopapular), dry skin, eczema, skin peeling, or blisters on the lips, eyes, or mouth. Patients were prophylactically prescribed cetirizine to prevent rash.

*Patients’ reported outcomes*. CAP/FUL also showed a positive impact on patients’ reported outcomes, delaying time to deterioration in EORTC QLQ-C30 global health status/quality of life (QoL) compared with the placebo/FUL arm (24.9 months with 95% CI 13.8 to not reached versus 12 months with 95% CI 10.2–15.7; HR 0.70, 95% CI 0.53–0.92). The two groups of patients did not show significant differences in time-to-deterioration of global QoL and the QLQ-BR23 breast module subscale scores. However, diarrhea was worse in the CAP/FUL group compared with the placebo/FUL group (HR 2.75, 95% CI 2.01–3.81) [46]. Although the trial was not powered for the symptom assessment with the Patient-Reported Outcomes version of the Common Terminology Criteria for Adverse Events (PRO-CTCAE), data showed that diarrhea (frequent or almost constant) was one-third more frequent in the CAP arm than in the control arm at the beginning of therapy and decreased at subsequent cycles. Rash, mouth or throat sores, itchy skin, and numbness or tingling in the hands or feet were other PRO-CTCAE-reported symptoms, but they were absent or mild in most patients in both groups throughout treatment. According to the Patient Global Impression of Treatment Tolerability (PGI-TT), most patients in both groups reported “not at all” or “a little bit” of bother from treatment side effects. Further analysis of the 78 Japanese patients showed that the safety profile of CAP/FUL did not differ from that recorded in the global population [35].

## 7. Post Marketing

To date, post-marketing data have come from small-size reports. Due to the preliminary nature of these real-world data, these findings should be interested with caution to avoid overinterpretation. A retrospective analysis of 34 HR+/HER2−/BC patients pretreated with CDK4/6i, T-DXd (44%), chemotherapy (44%), and alpelisib (38%) showed partial response in six patients and stable disease in six, with a mPFS of 3.5 months [37]. Grade 3 AEs were limited to diarrhea and hyperglycemia, each occurring in one patient. Data suggested that CAP could be an option for patients previously treated with alpelisib, with manageable toxicity. Some cases of capivasertib-associated diabetic ketoacidosis (DKA) with evidence of extreme insulin resistance have been reported so far [45]. DKA was managed with insulin infusion. CAP may also induce radiation recall dermatitis, which can be managed with corticosteroids, antibiotics, and switching to alpelisib [47].

Hofher and Clifton reported toxicity data for 29 patients with mutated BC treated with CAP/FUL, who had received an average of 3.6 lines of treatment (1–11) [38]. Overall, nine patients (36%) reported Grade 3 or higher side effects, including diarrhea (50%), rash (24%), nausea (24%), and hyperglycemia (24%). Unexpected side effects included acute kidney injury (6.8%), DRESS (3.4%), liver function tests (LFTs) 5 times the upper normal limits (6.8%), and colitis (3.4%). The median onset of diarrhea was 9 days (1–45 days).

## 8. Capivasertib Combination with Other Agents

The open-label BEECH trial evaluated the efficacy of CAP plus first-line weekly paclitaxel (PTX) in advanced or metastatic ER+/HER2−/BC and in a PIK3CA+ patient subpopulation [48]. In the phase Ib safety run-in (part A), 38 patients with advanced BC received PTX 90 mg/m^2^ (days 1, 8, and 15 of a 28-day cycle) with CAP twice daily under two intermittent, ascending-dose schedules, with safety as the primary endpoint to recommend a dose and schedule for part B. In the randomized, placebo-controlled, double-blind, phase II expansion (part B), 110 women with metastatic ER+/HER2−/BC were randomly assigned, stratified by PIK3CA mutation status, to receive PTX with either CAP or placebo. The primary endpoints for part B were PFS in the overall population and the PIK3CA+ subpopulation. CAP was well tolerated, and a 400 mg b.i.d. schedule on a 4-days-on/3-days-off schedule was selected in part A. In part B, mPFS in the overall population was 10.9 months (95% CI 8.3–12.4) with CAP versus 8.4 months (95% CI 8.2–10.8) with placebo (HR 0.80; *p* = 0.308). In the PIK3CA+ subpopulation, mPFS was 10.9 months with CAP versus 10.8 months with placebo (HR 1.11; *p* = 0.760). The most common Grade ≥ 3 adverse events in the CAP group were diarrhea, hyperglycemia, neutropenia, and maculopapular rash. Although manageable, Grade ≥ 3 adverse events (52%) were more frequent in the CAP group as compared to the placebo group (20%). Paclitaxel dose intensity was similar in both groups. CAP had no apparent impact on the tolerability or dose intensity of PTX. Adding CAP to weekly paclitaxel did not prolong PFS in the overall population or the PIK3CA+ subpopulation of advanced/metastatic ER+/HER2−/BC. Moreover, evidence suggests additional potential uses of CAP in other BC settings, including the triple-negative form. Besides the above reported negative results, the BEECH trial showed other key insights and limitations, such as the relatively small number of patients in specific sub-groups, i.e., the AKT1 (E17K) mutated one, which limited the statistical power. Despite these pitfalls, the BEECH trial was important in demonstrating that early on-treatment circulating tumor DNA (ctDNA) dynamics can act as a surrogate for PFS. This finding helped explain the lack of difference in PFS between the treatment arms, suggesting that earlier, more targeted interventions might be necessary, even if it did not immediately result in a positive, practice changing.

The CAPItello-290 trial enrolled 812 patients with previously untreated metastatic TNBC randomized to receive PTX 80 mg/m^2^ on day 1/3 weeks plus CAP 400 mg or placebo twice daily (days 2–5, weeks 1–3) [49]. The difference in median OS for the overall population was not significant in the overall and the PIK3CA/AKT1/PTEN-mutated population (31% of patients) (17.7 and 18.0 months with CAP/PTX and placebo-PTX, respectively (HR 0.92; 95% CI 0.78–1.08; *p* = 0.3239) or in patients with PIK3CA/AKT1/PTEN-altered cancers: 20.4 months in both arms (HR 1.05; 95% CI 0.77–1.43; *p* = 0.7602). On the other hand, mPFS in the overall population was longer in the CAP/PTX arm as compared to the placebo arm (5.6 versus 5.1 months; HR 0.72; 95% CI 0.61–0.84) and in patients with PIK3CA/AKT1/PTEN-altered cancer (7.5 versus 5.6 months; HR 0.70; 95% CI 0.52–0.95). The most frequent toxicity was Grade ≥ 3 diarrhea (12.7% versus 0.7%). The results of the CAPItello-290 study illustrate how a biologically rational combination can fail to translate into survival benefit in a heterogeneous, aggressive cancer such as TNBC underscoring the need for finer biomarker stratification, better understanding of resistance mechanisms, and more refined patient selection beyond broad pathway alteration testing. TNBC remains one of the most challenging cancers to treat due to the lack of known actionable biomarker targets, with chemotherapy-based regimens continuing to be the mainstay of treatment.

In the phase I, open-label, SERENA-1 trial, oral CAP 400 mg (4 days on, 3 days off) has been tested in combination with the next-generation oral selective estrogen receptor degrader and complete ER antagonist camizestrant 75 mg/day [50]. Patients were mostly pretreated with FUL and CDK4/6i. The safety profile was acceptable, with diarrhea (75.9%) and nausea (44.8%) being the most common adverse events. median progression-free survival was 8.3 months. Moreover, AKT inhibitors may positively interact with immunotherapy since AKT inhibition enhances the antitumor efficacy of immune checkpoint blockades and radiotherapy in a syngeneic BC preclinical model [51].

## 9. Resistance to Capivasertib

**Figure 3:**
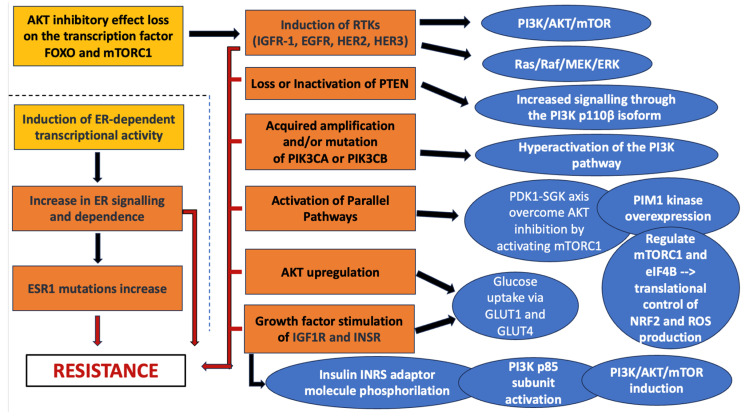
Resistance to targeted inhibitors of the PI3K/AKT/mTOR pathway in BC. Induction of receptor tyrosine kinase (RTK) including insulin growth factor-1 (IGFR-1), epidermal growth factor receptor (EGFR), and HER 2 and 3 may activate PI3K/AKT/mTOR and Ras/Raf/MEK/ERK, leading to resistance. Other mechanisms include loss or inactivation of PTEN, acquired amplification and/or mutation of PIK3CA or PIK3CB, which increase signaling through the PI3K p110β isoform and hyperactivation of the PI3K pathway, AKT up-regulation, which increases glucose uptake, and growth factor stimulation of IGF1R and INSR, which, once phosphorylated, activates the PI3K p85 subunit inducing PI3K/AKT/mTOR pathway.

Figure 3 depicts the main mechanisms underlying resistance to targeted inhibitors of the PI3K/AKT/mTOR pathway in BC. 

Hopcroft et al. examined how prior CDK4/6i treatment affects ER+BC cell function and response to CAP/FUL in BC cell lines [52]. In RB+ T47D and MCF7 cells, as well as in RB− palbociclib-resistant cells, ER pathway activity and ER-binding protein 1 (Greb-1) expression were reduced compared with naïve cells. PI3K-AKT pathway activation was also altered in RB+ cells, and CAP was less effective at lowering phosphorylated ribosomal protein S6 (pS6), a key marker of active mTOR signaling. In resistant cells, the combination was less effective at reducing cell cycle gene expression. Still, a consistent reduction in the S-phase cell fraction was observed in naïve and resistant cells. RB+ and RB− palbociclib-resistant cells responded to combination treatment, despite a modest decrease in relative efficacy. 

In tumors with *PIK3CA* alterations, heightened mTORC1-driven translation appears to confer innate resistance. Consequently, assessing mTORC1 activity could be a valuable addition to the current genetic selection approach for CAP. Sobsey et al. examined proteomic profiles associated with the CAP response in cancer cell lines and in 16 patients selected for genetic predisposition before treatment [53]. Patients were classified as having clinical benefit or no clinical benefit based on PFS > 12 weeks or <12 weeks, respectively. Proteins that differed between these groups were then measured in AKT1- or PIK3CA-altered BC cell lines with varying sensitivity to CAP. Although AKT1 and AKT2 levels did not differ significantly between the two groups, translational activity was higher in tumors from patients without clinical benefit. When quantified using validated LC-MRM-MS assays, the same proteins effectively differentiated between CAP-sensitive and -resistant cell lines. These findings support the idea that increased mTORC1-driven translation may be a resistance mechanism to CAP monotherapy. 

Loss of PTEN expression, whether through homozygous or hemizygous deletion, is common in PIK3CA-mutant ER+BC. We evaluated how reduced PTEN protein levels affect the efficacy of the AKT inhibitor capivasertib in tumors with PIK3CA alterations. In models with PIK3CA alterations and high PTEN protein levels, both PI3Kα and AKT inhibitors were effective. However, loss or partial reduction in PTEN expression decreased the effectiveness of PI3Kα inhibitors but did not impact capivasertib activity, whether used alone or in combination with FUL. The FOXO3a gene encodes a transcription factor that regulates apoptosis, cell proliferation, DNA repair, and the response to oxidative stress. It functions as a tumor suppressor and contributes to longevity.

Conversely, the FOXM1 gene encodes a transcription factor vital for cell cycle progression, DNA repair, cell division, and differentiation. It acts as a master regulator, activating genes involved in DNA replication and mitosis. FOXM1 is frequently overexpressed in cancer, promoting tumor growth, metastasis, and resistance to treatments. The effectiveness of CAP depends on FOXO3 and is linked to the suppression of FOXM1. Loss of FOXO3A reduces the response to CAP and increases FOXM1 levels. Prolonged exposure of ER+ breast cancer cells to CAP increases FOXM1 expression [54]. Reducing FOXM1 restores sensitivity to CAP, while overexpressing FOXM1 diminishes its effectiveness. Overall, these findings indicate that the AKT-FOXO3-FOXM1 pathway is crucial in determining how ER+ breast cancers with PIK3CA mutations respond to AKT inhibitors, regardless of PTEN status. Loss of FOXO3 can lead to resistance, and decreased FOXM1 levels may serve as a biomarker for treatment response.

The results of the SOLAR-1 and CAPItello-291 trials highlighted the benefit of the α-selective PI3K inhibitor alpelisib and CAP in patients with HR+/HER2−/BC harboring PIK3CA/AKT1/PTEN alterations. Sirico et al. retrospectively analyzed the predictive value of markers, including *PIK3CA*, *AKT*, and *PTEN* mutations; insulin levels; and FDG-PET/TC [11]. Only *PIK3CA* mutations (PIK3CA-mut) and AKT pathway alterations showed predictive value for CAP and alpelisib [11]. Given tumor heterogeneity, the authors suggested tailoring treatment based on *PIK3CA* mutational status in both metastatic tissue and blood at disease progression.

## 10. Pharmacoeconomics

The cost-effectiveness of CAP/FUL was analyzed using OS and PFS data from the CAPItello-291 trial, which were extrapolated to estimate long-term survival [55]. The primary outcome measure was the incremental cost–utility ratio (ICUR), which estimated the ICUR for CAP/FUL versus FUL alone to be $709,647 per quality-adjusted life-year (QALY) in the US. The authors concluded that CAP/FUL is unlikely to be a cost-effective option compared to single-agent FUL for HR+/BC from the perspective of the healthcare system in the US and China. Other scientists that compared the costs and efficacy of three treatment regimens using a Markov model came to the same findings [50,51]. When CAP was added to FUL for all patients, expenses increased by $410,765 and QALYs increased by 1.46 when compared to FUL alone. This resulted in an incremental cost effectiveness ratio of $280,854/QALY [56,57]. Therefore, it seems necessary to engage in negotiations over the pricing.

## 11. Discussion

**Figure 4:**
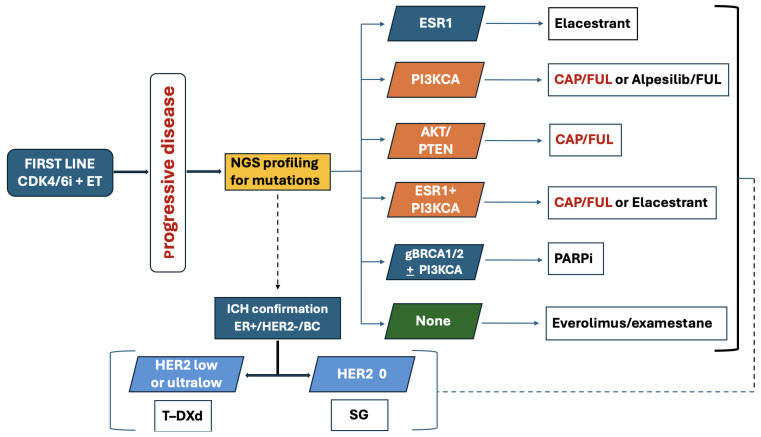
Proposed treatment algorithm for patients with HR+/HER2−/BC progressing after CDK4/4i plus endocrine therapy.

Endocrine therapy with CDK4/6i plus endocrine therapy is the standard first-line treatment for advanced or metastatic HR+/HER2− breast cancer. Despite durable benefit, most patients ultimately develop resistance, frequently driven by activation of the PI3K/AKT/mTOR pathway [58]. This has provided a strong rationale for the development of targeted therapies aimed at overcoming endocrine resistance. Figure 4 depicts the treatment algorithm for HR+/HER2−/BC progressing after CDK4/4i plus endocrine therapy based on molecular profiling and reassessment of hormonal and HER-2 status.

CAP has demonstrated robust preclinical and clinical activity in this setting. Some phase II–III trials, most notably CAPItello-291, showed a significant PFS improvement with CAP plus FUL compared with FUL alone. This benefit was largely confined to patients with PI3K/AKT/PTEN pathway-altered tumors, supporting biomarker-driven patient selection.

CAP expands the therapeutic options in this population of PI3K/AKT/PTEN pathway–altered tumors. Unlike alpelisib, which is limited to PIK3CA-mutated disease, capivasertib also targets *AKT1* and *PTEN* alterations. The safety profile of capivasertib is generally manageable, with diarrhea, rash, and hyperglycemia occurring early and responding to supportive measures and dose modifications [59]. Nevertheless, rare but severe metabolic events underscore the importance of careful patient selection and close monitoring. Ongoing studies are exploring CAP in combination strategies, including triplet regimens with CDK4/6i and ET, as well as with next-generation endocrine agents. Additionally, emerging translational data suggest that functional and proteomic biomarkers may further refine patient selection beyond genomic alterations alone.

In summary, CAP represents an effective option for patients with endocrine-resistant HR+/HER2−/BC harboring PI3K/AKT/PTEN alterations. Future research will clarify its optimal sequencing, combination strategies, and long-term clinical impact.

## Figures and Tables

**Table 1:** Pharmacokinetics, pharmacodynamics, indication, and dosing of capivasertib in breast cancer.

Therapeutic Class	Serine/Threonine Kinase AKT Inhibitor of All 3 Isoforms of Serine/Threonine Kinase AKT (*AKT1*, *AKT2*, and *AKT3*). Inhibits Phosphorylation of Downstream AKT Substrates		
Steady-state AUC	8069 h/ng/mL (37%)		
Cmax	1371 ng/mL (30%)		
Tmax	1–2 h		
Absolute bioavailability	29%		
High-fat or low-fat meal	No clinically meaningful differences		
Steady-state volume of distribution	1847 L (36%)		
Plasma protein binding	22%; Plasma-to-blood ratio 0.71		
Metabolism	CYP3A4 and UGT2B7 → check for drug interactions		
Clearance	Following a single radiolabeled oral dose of capivasertib 400 mg, mean total recovery was 45% from urine and 50% from feces		
Renal clearance	21% of total clearance		
Race/sex/body weight	No clinically significant differences in pharmacokinetics		
Mild to moderate renal impairment *	No clinically meaningful differences		
Moderate hepatic impairment **	Not fully cleared. No data on severe impairment		
Approval/indications	In combination with FUL for treatment of adults with advanced or metastatic HR+/HER2−/BC with 1 or more *PIK3CA*/*AKT1*/*PTEN* alterations (as determined by an FDA-approved test) following progression on at least 1 endocrine-based regimen in the metastatic setting or recurrence on or within 12 months of completing adjuvant therapy.		
Schedule	400 mg (two 200 mg tablets) twice daily, with or without food, for 4 days followed by 3 days off; continued until disease progression or intolerable toxicity occurs		
Main warnings	Adverse event	All grades (% cases)	Grade 3–4
	Diarrhea	77%	12%
	Skin	56%	15%
	Fatigue	38%	1.9%
	Nausea	35%	1.3%
	Vomiting	21%	1.9%
	Hyperglycemia	19%	1.9%
	Renal injury	11%	2.6%

* Creatinine clearance 30–89 mL/min; ** bilirubin greater than 1.5–3 times ULN and any AST.

**Table 2:** Main published studies of CAP plus FUL in hormone receptor-positive, HER-2-negative, advanced/metastatic breast cancer.

References	Patients	Treatment Arm	Number of Patients	ORR	Median PSF (Months)	Hazard Ratio (HR)	Median Overall Survival (Months)	
Jones et al. 2020 [31]	All	CAP/FUL	69	29.0%	10.3	HR 0.58 *p* = 0.0049	26.0	HR 0.59 *p* = 0.071
		PLA/FUL	71	8.0%	4.8		22.0	
	Mutated	CAP/FUL	28	Not reported	9.5	HR 0.59 *p* = 0.064 *	30.5	HR 0.53 *p* = 0.17
		PLA/FUL	31		5.2		28.7	
Howell et al. 2022 [32]	All	CAP/FUL	69	Not reported	10.3	HR 0.56 *p* = 0.0023	29.3	HR 0.66 *p* = 0.035
		PLA/FUL	71		4.8		23.4	
	Mutated	CAP/FUL	31	Not reported	12.8	HR 0.44 *p* = 0.0014	38.9	HR 0.46 *p* = 0.0047
		PLA/FUL	28		4.6		20.0	
Turner et al. 2023 [33]	All	CAP/FUL	355	22.9%	7.2	HR 0.60 *p* < 0.001	Not reached	HR 0.74
		PLA/FUL	353	12.2%	3.6			
	Mutated	CAP/FUL	155	28.8%	7.3	HR 0.50 *p* < 0.001	Not reached	HR 0.69
		PLA/FUL	134	9.7%	3.1			
Oliveira et al. 2024 [34]	All	CAP/FUL	355	Not reported	7.2	HR 0.60 *p* < 0.001	Not reached	HR 0.74
		PLA/FUL	353		3.6			
	Mutated	CAP/FUL	155	Not reported	7.3	HR 0.50 *p* < 0.001	Not reached	HR 0.69
		PLA/FUL	133		3.1			
	Wild type	CAP/FUL	200	Not reported	5.3	HR 0.79	Not reached	Not reported
		PLA/FUL	219		3.7			
Tokunaga et al. 2025 [35]	All	CAP/FUL	37	29.4%	13.9	HR 0.73	Not reported	Not reported
		PLA/FUL	41	22.0%	7.6			
	Mutated	CAP/FUL	19	27.8%	13.9	HR 0.65	Not reported	Not reported
		PLA/FUL	19	15.5%	9.1			
Hu et al. 2025 [36]	All	CAP/FUL	71	29.4%	6.9	HR 0.51	Not reached	Not reported
		PLA/FUL	63	8.3%	2.8			
	Mutated	CAP/FUL	21	Not reported	5.7	HR 0.41	Not reached	Not reported
		PLA/FUL	18		1.9			
Joshi et al. 2025 [37] (Retrospective)	Mutated	CAP/FUL	15	14.7%	3.5	Not reported	Not reached	Not reached
Hofher et al. 2025 [38] ** (Retrospective)	Mutated	CAP/FUL	29	Not reported	Not reported	Not reported	Not reported	Not reported
Khelifa et al. 2025 [39] (Metanalysis)	Not reported	Not reported	848	Not reported	Not reported	HR 0.61 *p* = 0.0124	Not reported	HR 0.68 *p* = 0.0209

* Not significant; ** toxicity data only.

## Data Availability

The original data presented in the study are openly available in medical literature (PubMed, Scopus).

## Abbreviations

The following abbreviations are used in this manuscript:

BC	Breast cancer
PFS	Progression-free survival
OS	Overall survival
HR+	Hormone receptor-positive
